# Exploring the Potential of ChatGPT-4 in Predicting Refractive Surgery Categorizations: Comparative Study

**DOI:** 10.2196/51798

**Published:** 2023-12-28

**Authors:** Aleksandar Ćirković, Toam Katz

**Affiliations:** 1 Care Vision Germany, Ltd Nuremberg Germany; 2 Department of Ophthalmology University Medical Center Hamburg-Eppendorf Hamburg Germany

**Keywords:** artificial intelligence, machine learning, decision support systems, clinical, refractive surgical procedures, risk assessment, ophthalmology, health informatics, predictive modeling, data analysis, medical decision-making, eHealth, ChatGPT-4, ChatGPT, refractive surgery, categorization, AI-powered algorithm, large language model, decision-making

## Abstract

**Background:**

Refractive surgery research aims to optimally precategorize patients by their suitability for various types of surgery. Recent advances have led to the development of artificial intelligence–powered algorithms, including machine learning approaches, to assess risks and enhance workflow. Large language models (LLMs) like ChatGPT-4 (OpenAI LP) have emerged as potential general artificial intelligence tools that can assist across various disciplines, possibly including refractive surgery decision-making. However, their actual capabilities in precategorizing refractive surgery patients based on real-world parameters remain unexplored.

**Objective:**

This exploratory study aimed to validate ChatGPT-4’s capabilities in precategorizing refractive surgery patients based on commonly used clinical parameters. The goal was to assess whether ChatGPT-4’s performance when categorizing batch inputs is comparable to those made by a refractive surgeon. A simple binary set of categories (patient suitable for laser refractive surgery or not) as well as a more detailed set were compared.

**Methods:**

Data from 100 consecutive patients from a refractive clinic were anonymized and analyzed. Parameters included age, sex, manifest refraction, visual acuity, and various corneal measurements and indices from Scheimpflug imaging. This study compared ChatGPT-4’s performance with a clinician’s categorizations using Cohen κ coefficient, a chi-square test, a confusion matrix, accuracy, precision, recall, *F*_1_-score, and receiver operating characteristic area under the curve.

**Results:**

A statistically significant noncoincidental accordance was found between ChatGPT-4 and the clinician’s categorizations with a Cohen κ coefficient of 0.399 for 6 categories (95% CI 0.256-0.537) and 0.610 for binary categorization (95% CI 0.372-0.792). The model showed temporal instability and response variability, however. The chi-square test on 6 categories indicated an association between the 2 raters’ distributions (χ²_5_=94.7, *P*<.001). Here, the accuracy was 0.68, precision 0.75, recall 0.68, and *F*_1_-score 0.70. For 2 categories, the accuracy was 0.88, precision 0.88, recall 0.88, *F*_1_-score 0.88, and area under the curve 0.79.

**Conclusions:**

This study revealed that ChatGPT-4 exhibits potential as a precategorization tool in refractive surgery, showing promising agreement with clinician categorizations. However, its main limitations include, among others, dependency on solely one human rater, small sample size, the instability and variability of ChatGPT’s (OpenAI LP) output between iterations and nontransparency of the underlying models. The results encourage further exploration into the application of LLMs like ChatGPT-4 in health care, particularly in decision-making processes that require understanding vast clinical data. Future research should focus on defining the model’s accuracy with prompt and vignette standardization, detecting confounding factors, and comparing to other versions of ChatGPT-4 and other LLMs to pave the way for larger-scale validation and real-world implementation.

## Introduction

### Background

Refractive surgery research has long strived to optimally precategorize patients to their respective ideal procedures, with the aim of minimizing the risk of complications like corneal ectasia based on initial measurements and findings while simultaneously maximizing refractive and subjective outcomes. Past advances in measurement data acquisition through modern examination techniques have led to the development of algorithms and indices that help in assessing risks and enhancing the workflow for refractive surgeons [1]. In particular, artificial intelligence (AI)–powered algorithms, such as machine learning approaches, have demonstrated promising results when applied to vast data sets generated by contemporary corneal examinations [2]. However, the selection of the most appropriate indices and algorithms from the broad spectrum of potential candidates and their optimal weighing of them with other clinical findings and one’s own past experience is still a matter of discussion and research. More recently, the emergence of large language models (LLMs) within the AI algorithms group, exemplified by Generative Pre-trained Transformer Version 4 and publicly accessible via the ChatGPT-4 chatbot (OpenAI LP) [3,4], has generated interest in their potential to serve as general AI, with the potential to assist across various disciplines [5-9]. Such models are not modeled to particular use-cases, but rather adapt to the current world knowledge database by using an enormous number of nodes and layers to process vast amounts of coherent texts, and as such can understand and answer to an exceptionally broad spectrum of instructions and questions, whose limits are yet to be explored [10]. Their potential lies within their worldwide low obstacle accessibility and generality, which enables them to assist in clinical or research data analysis by supporting the analysts as algorithm coders or as direct data analysts or decision-making supporters, addressing either laypersons or clinical assistant personnel [11,12]. Similar tests on patient vignettes have recently been performed in other domains of health care and other chatbot-accessible LLMs [13-16]. A preprint of a triage study on 10 vignettes containing patient complaints from the realm of ophthalmology without any further data to process showed promising, but also partially vague results [17]. Another diagnostic triage study found a worse performance of ChatGPT-4 as compared to ChatGPT-3.5 and Ada (Ada Health GmbH), a diagnosis app [18]. There is a multitude of other recent studies on this topic with similar designs which show some limited diagnostic ability for ChatGPT (OpenAI LP) [19-21]. For interactions with ChatGPT, a wide range of limitations is known so far, most notably logical and temporal inconsistency up to the point of nonsense in its answers, hallucinating, and prompt-dependency [10,19,22-25]. While it is known that the underlying Generative Pre-trained Transformer is a natural language processing model with a very large number of nodes and thus an LLM, and is using both unsupervised and supervised learning [7], the exact algorithms behind it remain undisclosed [19].

### Objectives and Scope

This exploratory study’s aim is to examine Generative Pre-trained Transformer Version 4’s capabilities in precategorizing refractive surgery patients via ChatGPT-4 and based on a limited set of parameters that are commonly used in everyday clinical practice and represent real-world decision-making. Its performance will be compared against our own categorization of the patients, performed by the refractive surgeon in charge of their treatment (author AĆ). The actual selection of the parameters to be used for categorization is a deliberate starting point and is motivated by being concise but sufficient for a clinician to decide upon their categorization as offered. In addition to patient baseline data such as refraction, visual acuity, and age, Scheimpflug imaging of the cornea is used for data acquisition, and indices from its software are used for its analysis. While not exhaustive in its scope, this study seeks to establish a foundation for more comprehensive research on ChatGPT-4’s utility in refractive surgery decision-making, and eventually also in other similar highly specialized fields. The aim and scope of this study does not include further detailed analysis of the variance of outputs different prompts would deliver; however, an initial modification of the prompts used to probe ChatGPT is inevitable to ascertain that reproducible and processable data are provided and will thus be noted, with the goal to process batches of data as large as possible per inquiry.

### Study Design

A sample of 100 patients will be used as a starting point for a statistical power calculation to determine whether this number will suffice for the planned analysis. As there are no similar predecessor studies available from this specific realm of medicine in scientific literature so far, we chose the daily clinical patient categorization as a starting point for a comparison, which will correspond to the actual surgery types offered in our clinic. The categories per patient examined will thus be (1) laser-assisted in situ keratomileusis (LASIK), (2) photorefractive keratectomy (PRK), (3) implantation of an intraocular collamer lens, (4) phacoemulsification and implantation of an intraocular lens, (5) no surgery due to a higher risk of corneal ectasia, and (6) no surgery due to other unspecified findings but still based on the variables used for this study. In addition, we plan to reevaluate the results with a more simplified categorization where the goal is just to discriminate between patients suitable for laser refractive surgery (LASIK/PRK combined) and all others, as the Scheimpflug indices are created and optimized to minimize hazards when planning laser refractive surgeries. As another recent study showed improved diagnostic scores for ChatGPT-4 when reducing the number of categories from 5 to 2 [26], we hypothesize that for a human rater, based on the findings from Scheimpflug imaging only and combined with the patients’ baseline data, this would be an easier assignment than to determine which surgery type offers the safest and best outcome, and thus ChatGPT may also perform better on this simpler task.

The primary objective of this study is to evaluate the correlation between a human rater and ChatGPT-4 in classifying refractive surgery patients into 2 sets of categories: a basic set and a more detailed set, to identify areas for future detailed investigation.

## Methods

### Clinical Data Collection

Patients’ data from June to August 2021 from our refractive clinic were anonymized and used for this investigation. To control for selection bias, consecutive patients treated by the author (AĆ) were used. Further, one eye per patient was randomly selected and used. The inclusion criteria were (1) first time visit of the patient, (2) subsequent microkeratome LASIK, femtosecond LASIK, transepithelial or alcohol-based PRK, intraocular collamer lens implantation or intraocular lens implantation, or diagnosis of a corneal ectatic disorder or predisposition, or noneligibility for any surgery based on the data. Exclusion criteria were (1) keratectasia at 1 year follow-up or (2) noneligibility for any refractive surgery but due to reasons not founded in the measurements (see Multimedia Appendix 1: flowchart of the inclusion or exclusion process of recruited patients). The included values in the initial evaluation were age, sex, manifest refraction in diopters (dpt) and degrees (°), best-corrected visual acuity (decimal), thinnest pachymetry (in µm), white-to-white diameter (in mm), anterior chamber depth (in mm), Ambrósio relational thickness maximum (ARTmax), average pachymetry progression index, Belin-Ambrósio-Display index (BAD), and the inferior-to-superior index. In addition, the respective categorization of the patients was noted.

### ChatGPT-4 Data Collection

The interactions with ChatGPT-4 [27] were performed with the May 24, 2023, version. The data were input as space-separated values with line breaks between data rows and was asked to be returned in the same manner. First, the maximum number of input rows yielding any result that could be processed further was noted and then used for all subsequent dialogues. We defined a result as “usable” for further analysis if the answer was returned in the tabular format that was expected and was not obviously nonsensical in relation to the question. It was tested whether zero shot prompting would lead to repeated usable return of data rows, and if not, one shot prompting would then subsequently be used, modified until the returned data were homogenous enough to be used for further evaluation. The data were subsequently sent to ChatGPT 12 times in total, limited by the time consumed by manually sending the data, and waiting for and analyzing the results.

### Statistical Power Calculations

From the initial data, relative probabilities for the respective categories for a statistical power calculation were calculated. The power calculation for Cohen κ was first performed. A modified approach was used due to its limitation to a maximum of 5 categories: for this estimate, the low-output categories “ECTASIA RISK” and “OTHER,” which both stand for noneligibility for surgery, were summarized, with the rationale that an underestimation of the error could be avoided by adding a significant number of cases to the calculated ones. For the lower boundary of agreement to be detected, a minimum κ value of 0.21 (fair agreement) was chosen. Another power calculation was performed for the chi-square test with 6 categories for a large effect size (w=0.50). A third power calculation was performed for Cohen κ reduced to 2 categories, this time with 0.41 (moderate agreement) as the lower boundary as we would expect a better agreement on fewer categories. Further, 0.05 was selected as the significance level for all calculations, and 0.80 as the level of power. In case the necessary number exceeded the first 100 patients that were included up to this point, the number would be adapted adequately, limited by time, budget, and scope of this study.

### Data Analysis

For all categorizations, the modes of ChatGPT’s categorizations were compared against the results of the clinician’s categorizations using Cohen κ coefficient, a confusion matrix and a chi-square test for comparison of the relative distributions of patients to categories by both raters. For constructing a CI for Cohen κ, 1000-fold bootstrapping of the data samples was used to find the 2.5 and 97.5 percentiles of κ. The metrics accuracy, precision, recall, and *F*_1_-score were calculated for ChatGPT’s categorizations. The comparisons were repeated with the data categorizations reduced to 2 groups: LASIK/PRK (1) or not (0), a method that has been used in one previous assessment of ChatGPT [26]. Here, an additional receiver operating characteristics area under the curve (AUC) score was calculated. In addition, if ChatGPT would not stay with 1 category for a data row throughout the 12 iterations, the mode value would be calculated for this data row, and for all data rows, correlations between the variables and the probability of a data row fluctuating between iterations would also be calculated. An additional analysis could then also present between which categories ChatGPT was most likely to fluctuate.

### Software

The statistical power calculations were performed with R (version 4.2.3; R Foundation for Statistical Computing). Statistical exploration of the data and comparisons of the groups were performed with Python (version 3.11.4; Python Software Foundation; see Multimedia Appendix 2: Python code used for data processing and Multimedia Appendix 3: R code used for statistical power calculations). ChatGPT-4 was used as a tool for development and debugging of the Python code, with primary development and proofreading remaining in our own hands. The Scheimpflug measurements were obtained with an Oculus Pentacam (OCULUS, Inc).

### Ethical Considerations

The patients’ records were anonymized for this study. All patients provided informed consent of the visit to our clinic, which included subsequent anonymized data analysis. This study was approved by the internal ethics review board of Care Vision Germany Ltd (CMLCB2023-01). No compensations were paid to the patients.

### Guidelines

In this study, we adhered to the Transparent Reporting of a Multivariable Prediction Model for Individual Prognosis or Diagnosis guidelines [28].

## Results

### Clinical Data Collection

Prior to sample size calculation, 55 female and 45 male consecutive patients were first included in this study. For this, a total of 167 consecutive patients (87 female and 80 male) were recruited. Of these, 121 were first time visiting patients. Of these, 17 did not undergo any refractive surgery procedures, and 4 were not eligible for surgery for other reasons beyond this study’s criteria. There were no cases of keratectasia among this study’s group. For a full flowchart of the inclusion or exclusion process, descriptive statistics and a correlation matrix of the original patients’ variables, see Multimedia Appendix 1: flowchart of the inclusion or exclusion process of recruited patients, Multimedia Appendix 4: descriptive statistics of the original variables with cutoff values to the top 20% (n=20) of ChatGPT judgmental instability, and Multimedia Appendix 5: correlation matrix of the original patients’ variables.

### Statistical Power Calculations

The modified power calculation for Cohen κ for 6 categories resulted in a minimum of 56 and for 2 categories in a minimum of 40 necessary subjects. The power calculation for the chi-square test resulted in a minimum of 52 cases. Thus, it was deemed sufficient to use the included 100 consecutive patients for subsequent analysis with ChatGPT.

### Data Analysis

ChatGPT returned nonsensical results if more than 50 data rows were input at once, thus the input was limited accordingly. Further, zero shot prompting did not give consistent results; thus, a prompt was prepared to be reused for one shot prompting the data rows repeatedly in a consistent manner (see Multimedia Appendix 6: recorded conversations with ChatGPT-4). For this, a patient that we would categorize as having a higher risk of ectasia was chosen and precategorized to establish a boundary ChatGPT could use.

Table 1 shows the comparison of categorizations as output by the clinician and ChatGPT.

**Table 1:** In total, 6-category categorizations by the clinician and ChatGPT (12 iterations) in comparison. Percentages are also absolute values (n=100).

Category	Clinician, %	ChatGPT, n (%)	ChatGPT mode, %
LASIK^a^	71	667 (56)	61
PRK^b^	9	267 (22)	21
ICL^c^	3	62 (5)	2
IOL^d^	11	121 (10)	12
ECTASIA RISK	4	76 (6)	4
OTHER	2	7 (1)	0

^a^LASIK: laser-assisted in situ keratomileusis.

^b^PRK: photorefractive keratectomy.

^c^ICL: intraocular collamer lens.

^d^IOL: intraocular lens.

The chi-square test showed a result of χ²_5_=94.7 with *P*<.001. Cohen κ coefficient was 0.399 (“minimal agreement” [29]), with a 95% CI 0.256-0.537. When comparing every respective iteration of ChatGPT’s categorization with the clinician, the coefficient’s range was 0.143-0.449. The highest percentage of agreements was found for category 1 (LASIK), followed by category 2 (PRK). A confusion matrix of the agreement between ChatGPT and the clinician is available in Multimedia Appendix 7: confusion matrix for 6-category categorizations by the clinician and ChatGPT (mode values). The accuracy of ChatGPT-4 for 6-category agreement was 0.68, the precision 0.75, the recall 0.68, and the *F*_1_-score 0.70 (Table 2).

**Table 2:** Overview of test results for the 6-category and the 2-category comparison between the clinician and ChatGPT-4.

Number of categories	Accuracy	Precision	Recall	*F*_1_-score	AUC^a^	Cohen κ
6	0.68	0.75	0.68	0.70	N/A^b^	0.399
2	0.88	0.88	0.88	0.88	0.79	0.610

^a^AUC: receiver operating characteristic area under the curve.

^b^N/A: not applicable.

The highest correlations between a variable and the probability of ChatGPT fluctuating its category estimate between the iterations were found for pachymetry and ARTmax (see Multimedia Appendix 8: Correlation of the variables’ categorization insecurity; the influence of the respective variables on the probability of ChatGPT changing categories). The highest count of fluctuations between categories occurred between categories 1 and 2, followed by 2 and 5 (see Multimedia Appendix 9: distribution of the most common fluctuations between 2 categories).

The cutoff values for each variable toward the top 20% (n=20) of all fluctuating data rows are available in Multimedia Appendix 4: descriptive statistics of the original variables with cutoff values to the top 20% (n=20) of ChatGPT judgmental instability. When analyzing the top 2 variations of fluctuating categorizations, the 3 variables with the highest respective correlations were pachymetry, anterior chamber depth, and sex for fluctuations between categories 1 and 2, and pachymetry, ARTmax, and BAD for fluctuations between categories 2 and 5 (for in-depth analyses of the fluctuations, see Multimedia Appendix 10: correlation of the categories with the probability of category change in ChatGPT’s iterations, Multimedia Appendix 11: correlations between the variables and ChatGPT fluctuating between categories 2 and 5, Multimedia Appendix 12: correlations between the variables and ChatGPT fluctuating between categories 1 and 2, and Multimedia Appendix 13: correlations between variables and ChatGPT fluctuating between 2 categories, sorted by absolute value).

On reanalysis of the output of both raters narrowed down to 2 categories, Cohen κ improved to 0.610 (“moderate agreement” [29]), with a 95% CI 0.372-0.792.

The output by category is shown in Table 3. A confusion matrix of the agreement between the raters is available in Multimedia Appendix 14: confusion matrix for binary categorizations of the clinician and ChatGPT.

**Table 3:** Binary categorizations by the clinician and ChatGPT (12 iterations) in comparison. Percentages are also absolute values (n=100).

Category	Clinician, %	ChatGPT, n (%)	ChatGPT mode, %
LASIK^a^/PRK^b^	80	934 (78)	82
NO SURGERY	20	266 (22)	18

^a^LASIK: laser-assisted in situ keratomileusis.

^b^PRK: photorefractive keratectomy.

Comparing the clinician to every respective ChatGPT iteration again, κ now had a range of 0.360-0.650. In the binary case, the accuracy was 0.88, precision 0.88, recall 0.88, the *F*_1_-score 0.88, and the AUC score was 0.79. A summary of the results is given in Table 2.

## Discussion

### Main Findings, Comparison to Previous Studies

In this study, we observed a significant association between the classifications made by a human rater and ChatGPT-4 in categorizing refractive surgery patients. This was more prominently evident in the basic binary categorization, but also—to a lesser degree—in the more intricate 6-category set. The agreement values of Cohen κ we obtained in this study are in line with or better than findings from other similar clinical validation studies [26,30]. The results particularly show an overlap with a recent study that observed higher accordance with human raters when narrowed down to 2 categories as compared to 5 [26]. There, the authors had found an increase of Cohen κ from 0.34 to 0.71, and an increase in *F*_1_-score from 0.461 to 0.821, with the AUC for the binary case at 0.846, an even greater increase than in our study. In 3 other exemplary studies, *F*_1_-score levels for ChatGPT’s diagnostic performance varied between 0.74 and 0.98 [31], 0.440 and 0.771 [32] (both ChatGPT-4), and 0.418 and 0.620 (ChatGPT-3) [33]. While it is controversial what constitutes a “good” *F*_1_-score, it is clear that any application in medicine must strive toward 1 to reduce risks for adverse events. Thus, our interpretation is that the relatively good *F*_1_-score and Cohen κ results at least warrant further inquiry. Further analysis of the data also showed a trend toward more “insecurity” in ChatGPT’s model in determining whether a patient should obtain PRK or LASIK, or discerning between PRK and rejecting surgery due to a higher risk for ectasia. In our experience, this overlaps with a human doctor’s daily struggle, and ChatGPT’s behavior reflects this in this undesirable aspect, too. The correlation of the variables with decision insecurity was not surprising, either—the pachymetry and the correlated ARTmax value, followed by the BAD, are generally considered good parameters for the detection of keratectasia [34,35]. As the correlation matrices show, some of the variables might have only a minor impact on the results or be redundant as per the correlation matrix of the variables, and could thus be omitted or replaced by more significant ones in follow-up studies.

### Limitations

#### Output Variance

However, it is essential to acknowledge the limitations of this study and the employed model. ChatGPT-4 did not provide stable outputs over multiple iterations of the data input. The variability in ChatGPT-4’s categorization between iterations, as evidenced by the range of Cohen κ coefficient, indicates the need for further investigation to define the borders of this behavior. This instability could be attributed to a probabilistic, random, or fuzzy element inherent in the AI model. A deeper understanding of this mechanism might enhance the accuracy of future models. Previous research has already demonstrated long-term changes of ChatGPT’s output [36]. Consequently, further investigation should include a detailed analysis of the short- and long-time temporal variations of ChatGPT’s output as well as careful analysis of the contributing factors. As long as these are unknown, the validity of this study’s results for health care applications remains low.

#### Usage of Patient Data Instead of Vignettes

Multiple problems arise from the fact that actual patient data and not specially designed vignettes were used for this study. First, ChatGPT had its strength in the more general binary task of recognizing patients suitable for LASIK/PRK; here, it showed the largest agreements with the clinician. On the other hand, it had more difficulties sorting those not suitable for LASIK/PRK correctly into the categories we offered, especially for low pachymetry and ARTmax values. Furthermore, one potential confounding factor here is that our study used only 1 clinician for comparison with ChatGPT, thus the result depends fully on his own professional knowledge and experience. Further inquiry with a larger number of clinicians could help validate the comparison better. We also cannot exclude that the human clinician may have considered more than just the data on hand as he decided on real-life patients, and ChatGPT lacked some human or other sensory data in comparison.

Second, our lack of knowledge about ChatGPT’s algorithms leaves room for speculation about further potential factors of influence like date and time the information is entered or the geographical location. Its algorithmic models could underly various biases, too.

Third, this study could also have benefitted from a detailed analysis of how modifying the prompting could influence the output, for example, by comparing the results between multiple iterations of zero-, one-, and few-shot prompting. Prompt design has already been a topic of research [22,23], but improving reproducibility will require some form of standardization.

Thus, in conclusion, future approaches will need to consider all these constraints when devising prompts and vignettes for a follow-up investigation.

#### Statistical Power, Bundling of Categories

A larger sample size would be needed to detect smaller effects than in this study. Another limitation is that the statistical power for the 6-category comparison with Cohen κ may have been overestimated due to the power calculation having been performed in a modified fashion on 5 categories beforehand.

Lastly, testing the binary categorizations was performed on bundled categorizations rather than reperforming the analysis with just 2 categories. For future studies, we recommend expanding the testing to also include a specific 2-category probing of ChatGPT, and also possibly other meaningful ways of categorizing the patients.

### Conclusions

This exploratory study found a promising correlation between one human rater and ChatGPT in refractive surgery, but cannot serve as more than an intermediary step to investigate this correlation on a broader basis. While the knowledge we have obtained so far may not justify the immediate application of ChatGPT in a clinical setting, it does warrant further inquiry. The potential use of ChatGPT as a precategorization tool seems plausible if we can solidify the boundaries of its limitations. The fact alone that an LLM, which has not been developed for such a highly specialized use-case, is potentially capable of solving problems in this setting, is remarkable and may change the general approach from trying to build highly specialized software to using general solutions as a form of general AI. As the progress between ChatGPT versions 3.5 and 4 was already significant, future investigations into new versions may also be promising. Furthermore, there is also a variety of other LLMs accessible to the public like ChatGPT-3.5, newer versions of ChatGPT-4, Google Bard and Bing AI (Microsoft Corp), which have also previously been tested for their usability in health care [13], as well as LLMs that are being developed for health care solely [16]. Testing these was beyond the scope of our study, but future comparisons to ChatGPT-4’s performance will be crucial to find the optimal LLM for health care applications. Another promising option for future analyses may be the customization of ChatGPT and possibly other LLMs for specific purposes that was recently introduced [27,37]. As the scientific knowledgebase on this topic grows, a process of standardization of approaches toward vignette-creation, prompting, and repeated probing of language-input chatbots seems inevitable. A recent proposal advocated for the development of a reporting standard for LLMs, which could help improve the quality of forthcoming analyses [38].

To our knowledge, this is the first study investigating the functionality of a (seemingly) general AI as a batch problem-solving tool in clinical refractive surgery. It may thus serve as a stepping stone for future research in this and similar specialized fields, paving the way for large-scale validation studies and real-world implementation research. The potential benefits of such a low-threshold AI for health care are substantial. The results of this study should, therefore, encourage further exploration into the application of LLMs in health care, particularly in decision-making processes that require a comprehensive understanding of a vast array of clinical data and that would previously have necessitated a highly specialized software, developed for very limited use cases only.

## Appendix

Multimedia Appendix 1 Flowchart of the inclusion/exclusion process of recruited patients.

Multimedia Appendix 2 Python code used for data processing.

Multimedia Appendix 3 R code used for statistical power calculations.

Multimedia Appendix 4 Descriptive statistics of the original variables with cut-off values to the top 20% (n=20) of ChatGPT judgmental instability. WtW = White-to-white corneal diameter. ACD = anterior chamber depth. ARTmax = Ambrósio relational thickness index, maximum value. avePPI = average Pachymetric Progression Index. BAD = Belin Ambrósio Display Index. Kmax = maximum steepness of the cornea. ISI = Inferior-superior-Index.

Multimedia Appendix 5 Correlation matrix of the original patients' variables.

Multimedia Appendix 6 Recorded conversations with ChatGPT-4.

Multimedia Appendix 7 Confusion matrix for six-category categorizations by the clinician and ChatGPT (mode values). Darker colours indicate higher agreement. Categories: 1 = LASIK, 2 = PRK, 3 = ICL, 4 = IOL, 5 = ECTASIA RISK, 6 = OTHER.

Multimedia Appendix 8 Correlation of the variables’ categorization insecurity. Red = negative, blue = positive correlation.

Multimedia Appendix 9 Distribution of the most common fluctuations between two categories. Categories: 1 = LASIK, 2 = PRK, 3 = ICL, 4 = IOL, 5 = ECTASIA RISK, 6 = OTHER.

Multimedia Appendix 10 Correlation of the categories with the probability of category change in ChatGPT's iterations.

Multimedia Appendix 11 Correlations between the variables and ChatGPT fluctuating between categories 2 and 5.

Multimedia Appendix 12 Correlations between the variables and ChatGPT fluctuating between categories 1 and 2.

Multimedia Appendix 13 Correlations between variables and ChatGPT fluctuating between two categories, sorted by absolute value.

Multimedia Appendix 14 Confusion matrix for binary categorizations of the clinician and ChatGPT. Darker blue shades indicate higher agreement.

## Abbreviations

AI: artificial intelligence
ARTmax: Ambrósio relational thickness maximum
AUC: area under the curve
BAD: Belin-Ambrósio-Display
LASIK: laser-assisted in situ keratomileusis
LLM: large language model
PRK: photorefractive keratectomy
