# Supplementary figures and images for: Exploring the Potential of ChatGPT-4 in Predicting Refractive Surgery Categorizations: Comparative Study
Source: JMIR Form Res. 2023 Dec 28;7:e51798. doi: 10.2196/51798 (PMC10784977; doi:10.2196/51798)

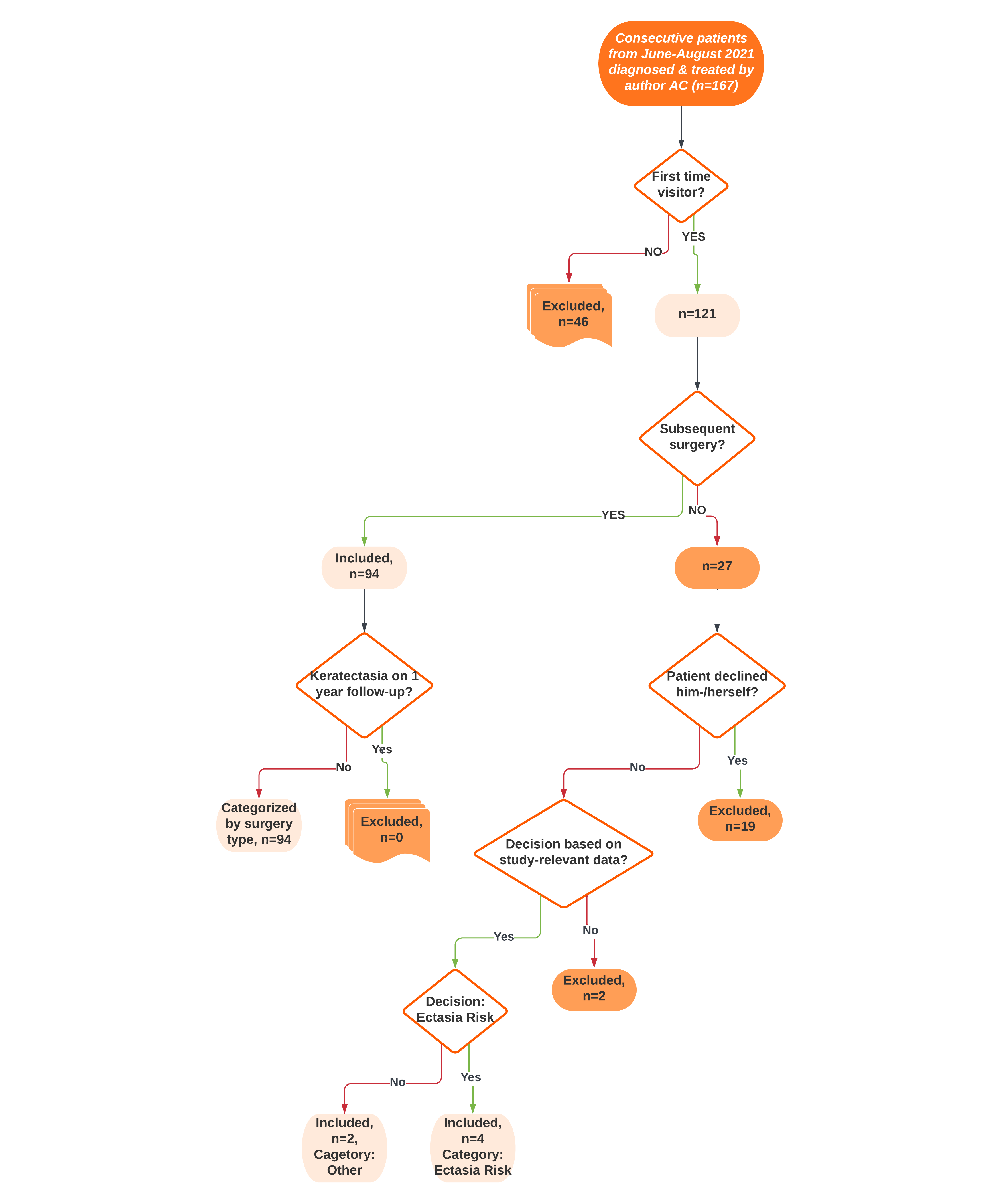

Supplement: Multimedia Appendix 1 [file formative_v7i1e51798_app1.png]

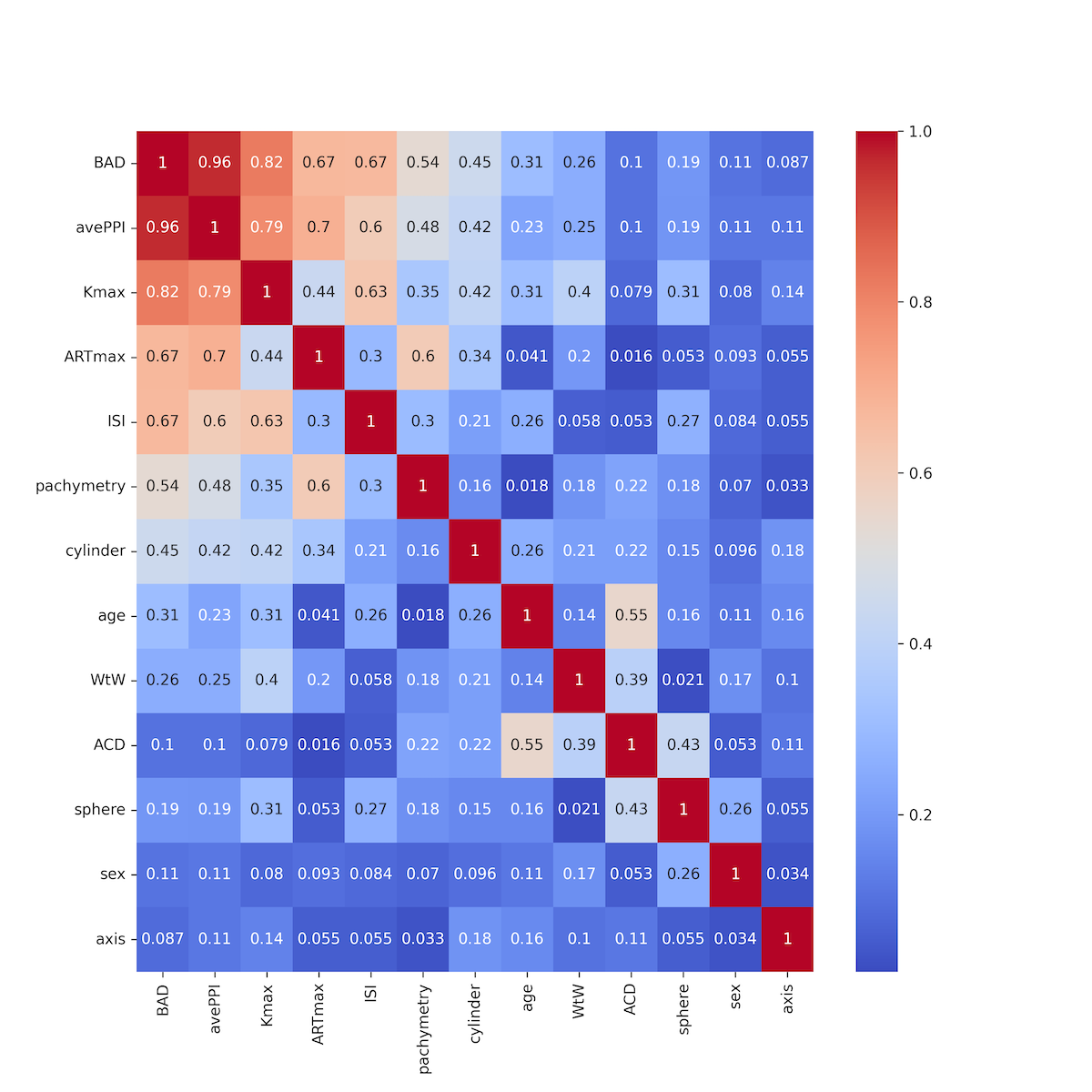

Supplement: Multimedia Appendix 5 [file formative_v7i1e51798_app5.png]

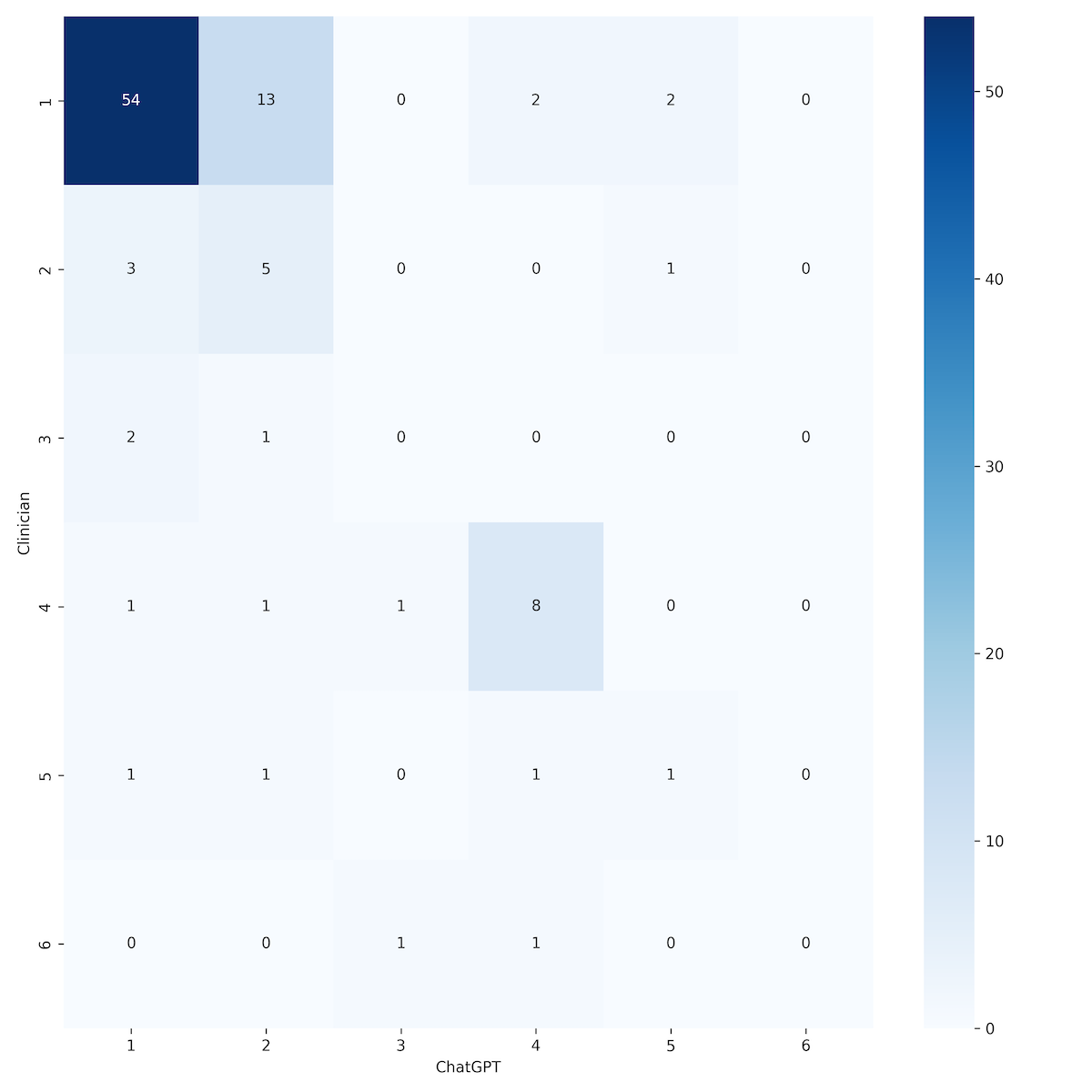

Supplement: Multimedia Appendix 7 [file formative_v7i1e51798_app7.png]

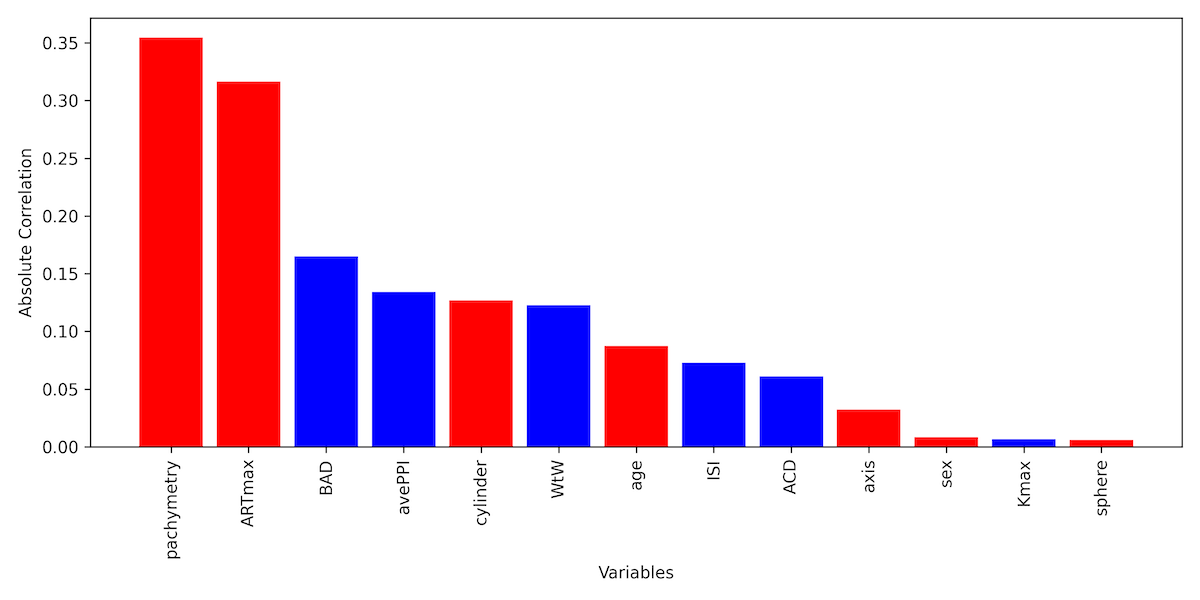

Supplement: Multimedia Appendix 8 [file formative_v7i1e51798_app8.png]

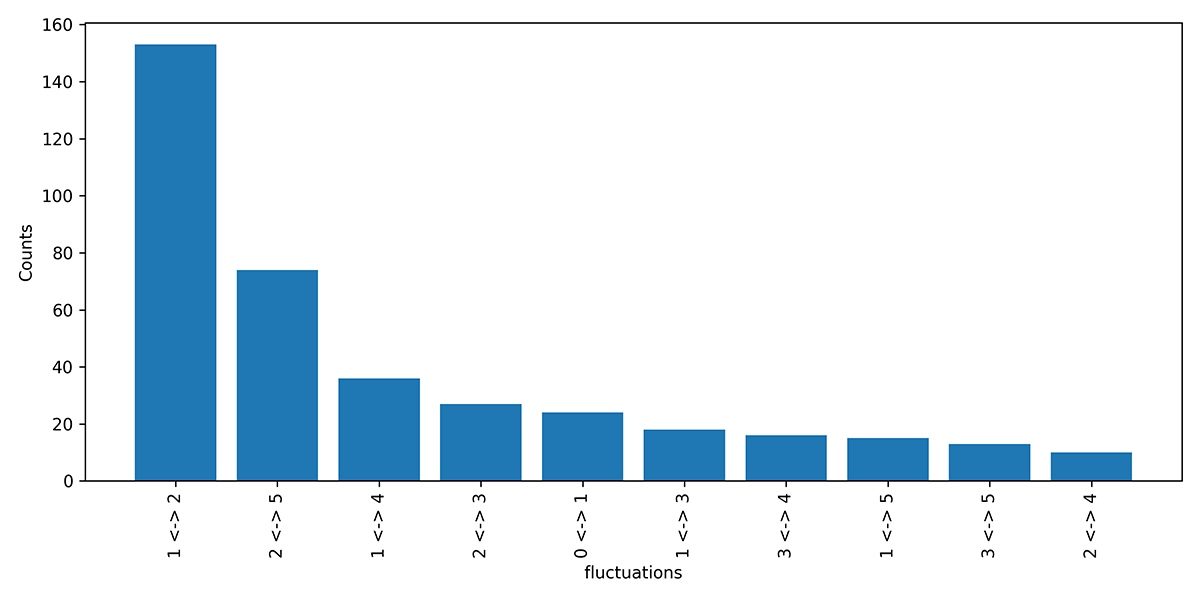

Supplement: Multimedia Appendix 9 [file formative_v7i1e51798_app9.png]

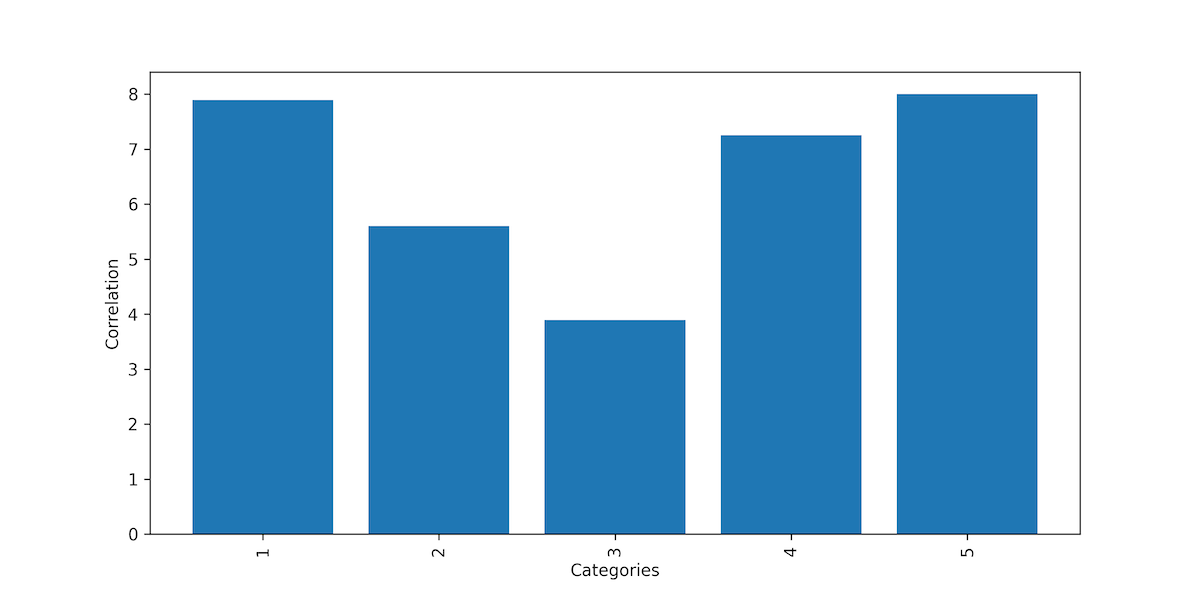

Supplement: Multimedia Appendix 10 [file formative_v7i1e51798_app10.png]

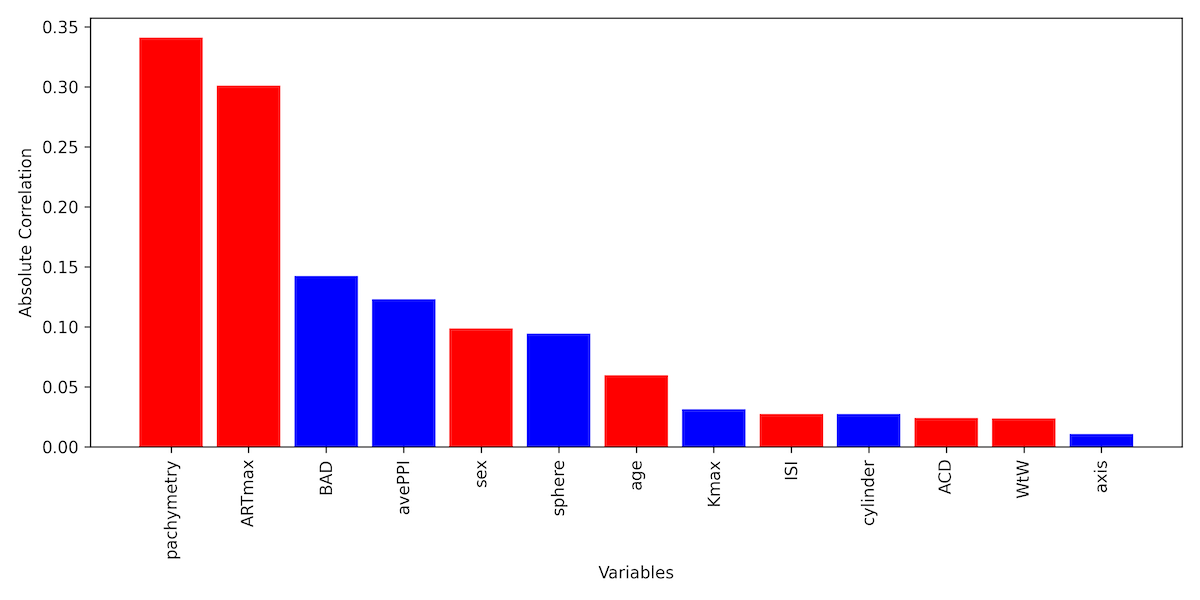

Supplement: Multimedia Appendix 11 [file formative_v7i1e51798_app11.png]

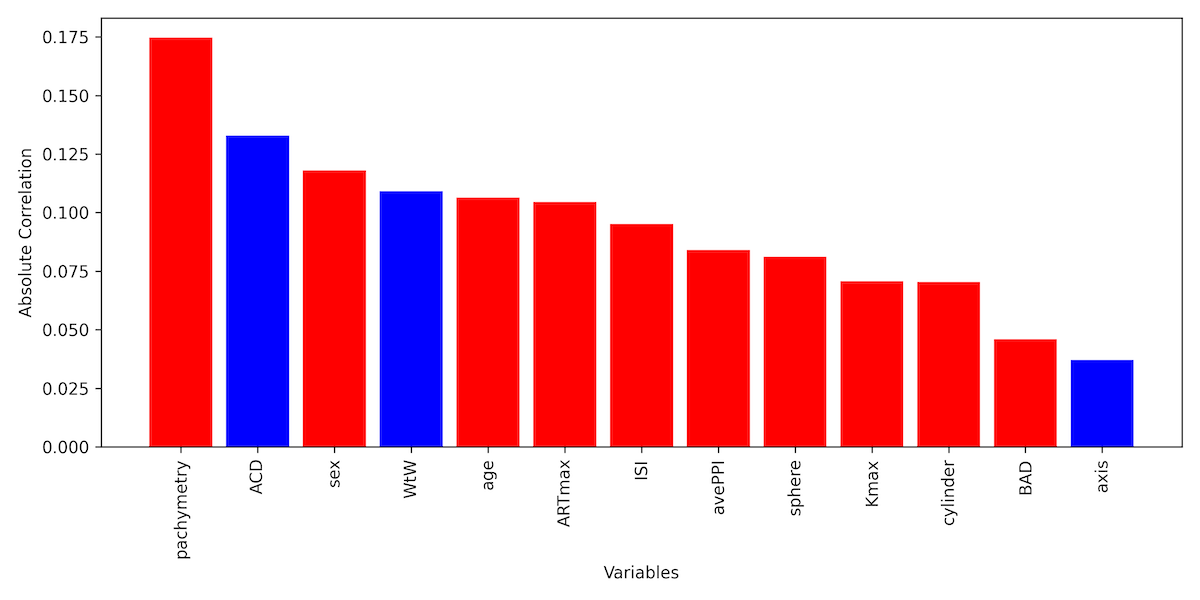

Supplement: Multimedia Appendix 12 [file formative_v7i1e51798_app12.png]

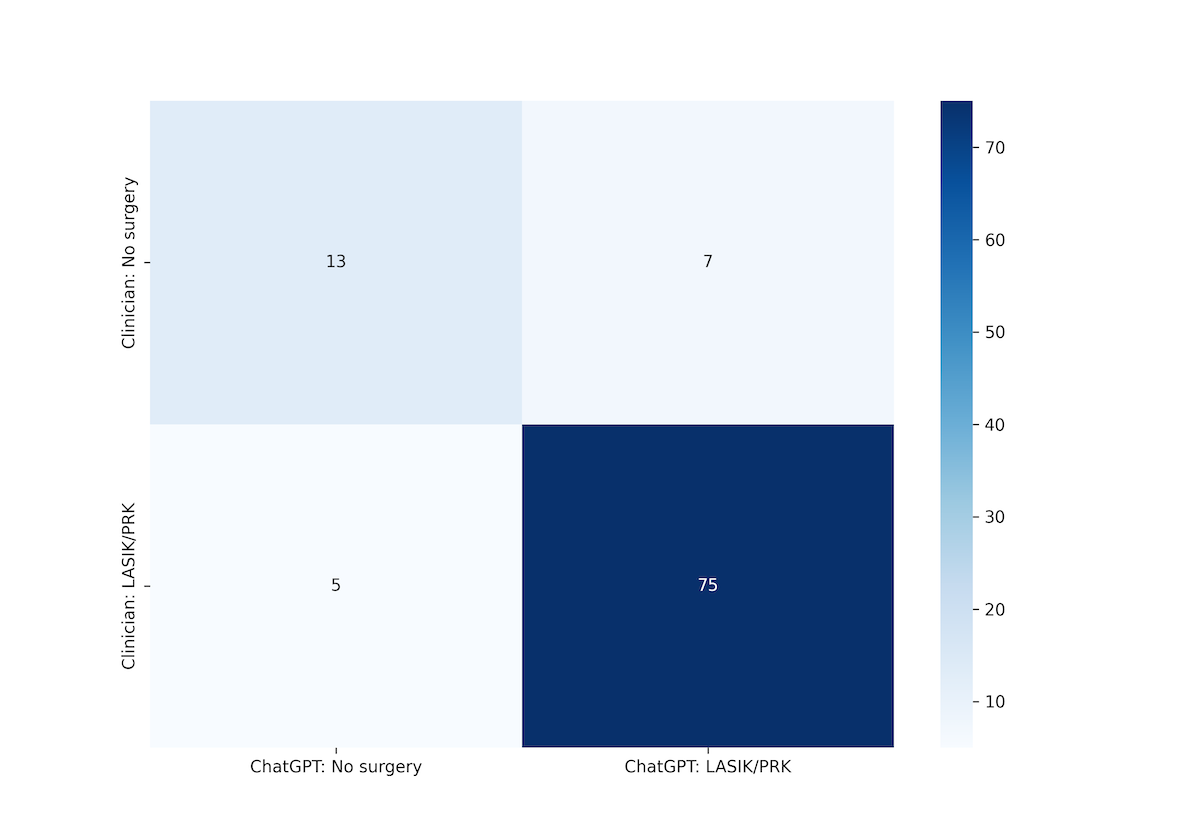

Supplement: Multimedia Appendix 14 [file formative_v7i1e51798_app14.png]
