# Supplementary material for: Exploring the Potential of ChatGPT-4 in Predicting Refractive Surgery Categorizations: Comparative Study
Source: JMIR Form Res. 2023 Dec 28;7:e51798. doi: 10.2196/51798 (PMC10784977; doi:10.2196/51798)
Supplement: Multimedia Appendix 2 [file formative_v7i1e51798_app2.docx]

Python code

# Print the category correlation

print('\nCorrelation of the categories with Categorization Instability:')

print(category_correlation)

# Create a bar plot for the correlation of the categories with data stability

plt.figure(figsize=(10, 5))

plt.bar(category_correlation.index.astype(int), category_correlation.values)

plt.title('Correlation of the Categories with Categorization Instability')

plt.xlabel('Categories')

plt.ylabel('Correlation')

import pandas as pd

import numpy as np

import matplotlib.pyplot as plt

import seaborn as sns

from scipy import stats

# Set pandas display options

pd.set_option('display.max_rows', None)

pd.set_option('display.max_columns', None)

pd.set_option('display.width', None)

pd.set_option('display.max_colwidth', None)

# Load the data

data = pd.read_csv('/Users/aleksandarcirkovic/Downloads/data.csv', decimal=',')

# Convert 'f' and 'm' to 0 and 1

data['sex'] = data['sex'].map({'f': 0, 'm': 1})

# Calculate the number of AI classification changes for each data row

data['fluctuations'] = data.iloc[:, 15:27].apply(lambda x: x.diff().abs().sum(), axis=1)

# Sort the data by the number of fluctuations

data_sorted = data.sort_values(by='fluctuations')

# Separate the data into the top 20% most fluctuating and the 20% most stable

fluctuating_data = data_sorted.tail(int(len(data_sorted) * 0.2))

stable_data = data_sorted.head(int(len(data_sorted) * 0.2))

# Calculate the statistical descriptions excluding the "Classification doctor 1" column

fluctuating_stats = fluctuating_data.drop(columns='Classification doctor').describe()

stable_stats = stable_data.drop(columns='Classification doctor').describe()

# Print the statistical descriptions

print('Fluctuating data statistics:')

print(fluctuating_stats.transpose())

print('\nStable data statistics:')

print(stable_stats.transpose())

# Calculate the absolute correlation of the variables with the stability and sort in descending order

abs_correlation_sorted = data_sorted.iloc[:, 1:14].corrwith(data_sorted['fluctuations']).abs().sort_values(ascending=False)

# Use the sorted index to reorder the original correlation series

correlation = data_sorted.iloc[:, 1:14].corrwith(data_sorted['fluctuations']).loc[abs_correlation_sorted.index]

# Print the correlation

print('\nCorrelation of the variables with Categorization Instability:')

print(correlation)

# Melt the AI classifications and calculate the mean number of fluctuations for each category

melted = data_sorted.iloc[:, 15:27].melt(var_name='AI_classification', value_name='category')

melted['fluctuations'] = data_sorted['fluctuations']

category_correlation = melted.groupby('category')['fluctuations'].mean().sort_values()

plt.xticks(rotation=90)

plt.savefig('/Users/aleksandarcirkovic/Downloads/figures/correlation_categories.png', dpi=300)

plt.show()

plt.close()

# Calculate the cut-off values for AI categorization insecurity for each variable

cutoff_values = {}

cutoff_indicators = {}

for variable in correlation.index:

if correlation[variable] > 0:

cutoff_values[variable] = data_sorted[variable].quantile(0.75)

cutoff_indicators[variable] = 'Higher values lead to more fluctuating'

else:

cutoff_values[variable] = data_sorted[variable].quantile(0.25)

cutoff_indicators[variable] = 'Lower values lead to more fluctuating'

print('\nCut-off values for AI categorization insecurity:')

for variable, cutoff in cutoff_values.items():

print(f'{variable}: {cutoff}, {cutoff_indicators[variable]}')

# Create a bar plot for the absolute correlation of the variables with data stability

plt.figure(figsize=(10, 5))

bars = plt.bar(correlation.index, correlation.abs())

plt.title('Absolute Correlation of the Variables with Categorization Instability')

plt.xlabel('Variables')

plt.ylabel('Absolute Correlation')

plt.xticks(rotation=90)

# Color the bars based on the direction of the correlation

for bar, value in zip(bars, correlation):

if value < 0:

bar.set_color('red')

else:

bar.set_color('blue')

plt.tight_layout()

plt.savefig('/Users/aleksandarcirkovic/Downloads/figures/correlation_variables.png', dpi=300)

plt.show()

plt.close()

# Calculate and print the descriptive statistics of the original variables

original_variables_stats = data_sorted.iloc[:, 1:14].describe()

print('\nDescriptive statistics of the original variables:')

print(original_variables_stats.transpose())

# Calculate the fluctuations between categories for each row

fluctuations = data_sorted.iloc[:, 15:27].apply(lambda x: x.diff(), axis=1)

# Create a new DataFrame to store the fluctuations between categories

fluctuations_df = pd.DataFrame(columns=['Category1', 'Category2'])

# Populate the DataFrame with the fluctuations

for i in range(fluctuations.shape[0]):

for j in range(fluctuations.shape[1] - 1):

if data_sorted.iloc[i, j + 15] != data_sorted.iloc[i, j + 17]: # Only consider a fluctuation if the category changes

temp_df = pd.DataFrame({'Category1': [min(int(data_sorted.iloc[i, j + 15]), int(data_sorted.iloc[i, j + 17]))],

'Category2': [max(int(data_sorted.iloc[i, j + 15]), int(data_sorted.iloc[i, j + 17]))]})

fluctuations_df = pd.concat([fluctuations_df, temp_df], ignore_index=True)

# Calculate the number of occurrences of each fluctuation

fluctuation_counts = fluctuations_df.groupby(['Category1', 'Category2']).size().reset_index(name='Counts')

# Sort the fluctuations by the number of occurrences and take the top 10

top_fluctuations = fluctuation_counts.sort_values(by='Counts', ascending=False).head(10)

# Print the most common fluctuations

print('\nMost common fluctuations between categories:')

print(top_fluctuations)

# Create a bar plot for the most common fluctuations

plt.figure(figsize=(10, 5))

plt.bar(top_fluctuations.apply(lambda x: f'{x.Category1} <-> {x.Category2}', axis=1), top_fluctuations['Counts'])

plt.title('Most Common Fluctuations Between Categories')

plt.xlabel('Fluctuations')

plt.ylabel('Counts')

plt.xticks(rotation=90)

plt.tight_layout()

plt.savefig('/Users/aleksandarcirkovic/Downloads/figures/most_common_fluctuations.png', dpi=300)

plt.show()

plt.close()

# Print the statistical descriptions in CSV format

print('Fluctuating data statistics:')

print(fluctuating_stats.to_csv())

print('\nStable data statistics:')

print(stable_stats.to_csv())

# Print the correlation in CSV format

print('\nCorrelation of the variables with Categorization Instability:')

print(correlation.to_frame().to_csv())

# Print the category correlation in CSV format

print('\nCorrelation of the categories with Categorization Instability:')

print(category_correlation.to_frame().to_csv())

# Print the cut-off values in CSV format

print('\nCut-off values for AI categorization instability:')

print(pd.Series(cutoff_values, name='Cut-off values').to_frame().to_csv())

# Print the descriptive statistics of the original variables in CSV format

print('\nDescriptive statistics of the original variables:')

print(original_variables_stats.to_csv())

from sklearn.metrics import cohen_kappa_score

import seaborn as sns

# Calculate the AI classification mode

data['AI_mode'] = data.iloc[:, 15:27].mode(axis=1)[0]

# Calculate Cohen's Kappa for comparison between "Classification doctor 1" and the mode AI classification

kappa = cohen_kappa_score(data['Classification doctor'], data['AI_mode'])

print(f"Cohen's Kappa for comparison between 'Classification doctor 1' and the mode AI classification: {kappa}")

# Calculate Cohen's Kappa for comparison between "Classification doctor 1" and every AI iteration separately

kappas = [cohen_kappa_score(data['Classification doctor'], data.iloc[:, i]) for i in range(15, 27)]

print("Cohen's Kappa for comparison between 'Classification doctor 1' and every AI iteration separately:")

print(kappas)

# Calculate min, max, and std for the values of Cohen's Kappa

print(f"Min: {min(kappas)}")

print(f"Max: {max(kappas)}")

print(f"Std: {np.std(kappas)}")

from sklearn.metrics import confusion_matrix

import seaborn as sns

# Calculate the confusion matrix

cm = confusion_matrix(data['Classification doctor'], data['AI_mode'])

# Create a heatmap from the confusion matrix

plt.figure(figsize=(10, 10))

sns.heatmap(cm, annot=True, fmt='d', cmap='Blues', xticklabels=range(1, 7), yticklabels=range(1, 7))

plt.xlabel('ChatGPT')

plt.ylabel('Clinician')

plt.title('Confusion Matrix: ChatGPT vs Clinician')

plt.tight_layout()

plt.savefig('/Users/aleksandarcirkovic/Downloads/figures/confusion_matrix.png', dpi=300)

plt.show()

# Print the most common fluctuations in CSV format

print('\nMost common fluctuations between categories:')

print(top_fluctuations.to_csv(index=False))

print(data['AI_mode'])

ai_mode_counts = data['AI_mode'].value_counts()

print(ai_mode_counts)

ai_mode_counts = data['AI_mode'].value_counts(sort=False)

print(ai_mode_counts)

# Convert the doctor's ratings into binary form

data['doctor_binary'] = data['Classification doctor'].apply(lambda x: 1 if x in [1, 2] else 0)

# Convert the AI mode into binary form

data['AI_mode_binary'] = data['AI_mode'].apply(lambda x: 1 if x in [1, 2] else 0)

# Calculate Cohen's Kappa for comparison between the binary doctor's ratings and the binary AI mode

kappa_binary = cohen_kappa_score(data['doctor_binary'], data['AI_mode_binary'])

print(f"Cohen's Kappa for comparison between the binary doctor's ratings and the binary AI mode: {kappa_binary}")

from sklearn.metrics import confusion_matrix

import seaborn as sns

# Calculate the confusion matrix

binary_confusion_matrix = confusion_matrix(data['doctor_binary'], data['AI_mode_binary'])

# Create a DataFrame from the confusion matrix for easier plotting

binary_confusion_df = pd.DataFrame(binary_confusion_matrix,

index=['Clinician: No surgery', 'Clinician: LASIK/PRK'],

columns=['ChatGPT: No surgery', 'ChatGPT: LASIK/PRK'])

# Plot the confusion matrix

plt.figure(figsize=(10, 7))

sns.heatmap(binary_confusion_df, annot=True, fmt='d', cmap='Blues')

plt.title('Confusion Matrix: Clinician\'s Ratings vs ChatGPT')

plt.savefig('/Users/aleksandarcirkovic/Downloads/figures/confusion_matrix_2.png', dpi=300)

plt.show()

from sklearn.metrics import cohen_kappa_score

from sklearn.utils import resample

# Initialize a list to store the bootstrap sample kappa scores

bootstrap_kappa_scores = []

# Perform bootstrapping

for _ in range(1000):

# Create a bootstrap sample

bootstrap_sample = data.sample(n=len(data), replace=True)

# Calculate and store the kappa score for the bootstrap sample

bootstrap_kappa_scores.append(cohen_kappa_score(bootstrap_sample['doctor_binary'], bootstrap_sample['AI_mode_binary']))

# Calculate the 95% confidence interval for the kappa scores

kappa_ci_lower = np.percentile(bootstrap_kappa_scores, 2.5)

kappa_ci_upper = np.percentile(bootstrap_kappa_scores, 97.5)

print(f"95% confidence interval for Cohen's Kappa: ({kappa_ci_lower:.3f}, {kappa_ci_upper:.3f})")

from sklearn.metrics import cohen_kappa_score

# Initialize a list to store the kappa scores

kappa_scores = []

# Loop over each AI iteration

for i in range(15, 27):

# Convert the AI categories to binary form

ai_binary = data.iloc[:, i].apply(lambda x: 1 if x in [1, 2] else 0)

# Calculate and store the kappa score

kappa_scores.append(cohen_kappa_score(data['doctor_binary'], ai_binary))

# Calculate min, max, and std for the values of Cohen's Kappa

print(f"Min: {min(kappa_scores)}")

print(f"Max: {max(kappa_scores)}")

print(f"Std: {np.std(kappa_scores)}")

# Calculate the correlation matrix

corr_matrix = data_sorted.iloc[:, 1:14].corr().abs()

# Calculate the sum of the absolute correlation values for each variable

corr_sum = corr_matrix.sum().sort_values(ascending=False)

# Reorder the correlation matrix

sorted_corr_matrix = corr_matrix.loc[corr_sum.index, corr_sum.index]

# Create a heatmap of the sorted correlation matrix

plt.figure(figsize=(10, 10))

sns.heatmap(sorted_corr_matrix, cmap='coolwarm', annot=True)

plt.title('Correlation Matrix of the Patient Measurements')

plt.savefig('/Users/aleksandarcirkovic/Downloads/figures/sorted_correlation_heatmap.png', dpi=300)

plt.show()

plt.close()

from scipy.stats import chi2_contingency

# Create a contingency table

contingency_table = pd.crosstab(data['Classification doctor'], data['AI_mode'])

# Perform chi-squared test

chi2, p_value, dof, expected = chi2_contingency(contingency_table)

print(f"Chi-Squared: {chi2}")

print(f"P-value: {p_value}")

print(f"Degrees of Freedom: {dof}")

# Create a contingency table

contingency_table_binary = pd.crosstab(data['doctor_binary'], data['AI_mode_binary'])

def calculate_correlation(data, cat1, cat2):

# Create a new column for fluctuations between the two categories

data['fluctuations'] = (data.iloc[:, 15:27].diff(axis=1).abs() == abs(cat1 - cat2)).sum(axis=1)

# Calculate the correlations of the variables with the ratio of "fluctuating" rows

correlation = data.iloc[:, 1:14].apply(lambda x: x.corr(data['fluctuations']))

return correlation

# Calculate the correlations of the variables with the ratio of "fluctuating" rows between two specific categories

correlation_2_5 = calculate_correlation(data, 2, 5)

correlation_1_2 = calculate_correlation(data, 1, 2)

# Sort the correlations

correlation_2_5_sorted = correlation_2_5.sort_values(key=abs)

correlation_1_2_sorted = correlation_1_2.sort_values(key=abs)

# Output the correlations as tab-separated tables

print("Correlations for Fluctuations Between Categories 2 and 5:")

print(correlation_2_5_sorted.to_string(), "\n")

print("Correlations for Fluctuations Between Categories 1 and 2:")

print(correlation_1_2_sorted.to_string())

def create_chart(correlations, title, filename):

plt.figure(figsize=(10, 5))

bars = plt.bar(correlations.index, correlations.abs())

plt.title(title)

plt.xlabel('Variables')

plt.ylabel('Absolute Correlation')

plt.xticks(rotation=90)

# Color the bars based on the direction of the correlation

for bar, value in zip(bars, correlations):

if value < 0:

bar.set_color('red')

else:

bar.set_color('blue')

plt.tight_layout()

plt.savefig(filename, dpi=300)

plt.show()

# Calculate the correlations

correlation_2_5 = calculate_correlation(data, 2, 5)

correlation_1_2 = calculate_correlation(data, 1, 2)

# Sort the correlations

correlation_2_5_sorted = correlation_2_5.sort_values(key=np.abs, ascending=False)

correlation_1_2_sorted = correlation_1_2.sort_values(key=np.abs, ascending=False)

# Create the charts

create_chart(correlation_2_5_sorted, 'Correlations for Fluctuations Between Categories 2 and 5', '/Users/aleksandarcirkovic/Downloads/figures/correlations_2_5.png')

create_chart(correlation_1_2_sorted, 'Correlations for Fluctuations Between Categories 1 and 2', '/Users/aleksandarcirkovic/Downloads/figures/correlations_1_2.png')

# Function to calculate metrics

def calculate_metrics(y_true, y_pred, label):

print(f"Metrics for {label}:")

print(f"Accuracy: {accuracy_score(y_true, y_pred)}")

print(f"Precision: {precision_score(y_true, y_pred, average='weighted', zero_division=1)}")

print(f"Recall: {recall_score(y_true, y_pred, average='weighted')}")

print(f"F1 Score: {f1_score(y_true, y_pred, average='weighted')}")

if len(y_true.unique()) == 2: # Check for binary classification

print(f"AUC Score: {roc_auc_score(y_true, y_pred)}")

print("------")

# Calculate metrics for 6-category classification

calculate_metrics(data.iloc[:, 14], data['AI_mode'], '6-category classification')

# Calculate metrics for 2-category classification

calculate_metrics(data['doctor_binary'], data['AI_mode_binary'], '2-category classification')
