# Supplementary material for: Exploring the Potential of ChatGPT-4 in Predicting Refractive Surgery Categorizations: Comparative Study
Source: JMIR Form Res. 2023 Dec 28;7:e51798. doi: 10.2196/51798 (PMC10784977; doi:10.2196/51798)
Supplement: Multimedia Appendix 3 [file formative_v7i1e51798_app3.docx]

Statistical Power Calculations

R 4.2.3 for Mac

> library(kappaSize)

> Power5Cats(0.01, 0.21, c(0.71, 0.09, 0.03, 0.11, 0.06), raters=2, alpha=0.05, power=0.8)

A minimum of 56 subjects are required for this study of interobserver agreement.

Warning: At least one expected cell count is less than five.

Warning: At least one expected cell count is less than five.

> library(pwr)

> pwr.chisq.test(w=0.5, N=NULL, df=5, sig.level = 0.05, power = 0.8)

Chi squared power calculation

w = 0.5

N = 51.31043

df = 5

sig.level = 0.05

power = 0.8

NOTE: N is the number of observations

> library(irr)

> N.cohen.kappa(0.8, 0.8, 0.41, 0, 0.05, 0.8, twosided=FALSE)

[1] 40
