# Supplementary material for: Exploring the Potential of ChatGPT-4 in Predicting Refractive Surgery Categorizations: Comparative Study
Source: JMIR Form Res. 2023 Dec 28;7:e51798. doi: 10.2196/51798 (PMC10784977; doi:10.2196/51798)
Supplement: Multimedia Appendix 4 [file formative_v7i1e51798_app4.docx]

|  | mean | std | min | max | cut-off to instable top 20% | |
| --- | --- | --- | --- | --- | --- | --- |
| age | 34.6 | 10 | 20 | 67 | < | 27.5 |
| pachymetry | 546.9 | 37 | 408 | 655 | < | 527.5 |
| sphere | -1.6 | 3.4 | -10 | 7.3 | < | -4.0 |
| cylinder | -1.3 | 1.1 | -5.8 | 0 | < | -1.8 |
| axis | 71.5 | 61.9 | 0 | 179 | < | 14.8 |
| WtW | 11.9 | 0.3 | 11 | 12.8 | > | 12.1 |
| ACD | 3.1 | 0.4 | 2.3 | 3.9 | > | 3.3 |
| ARTmax | 450.1 | 85.7 | 81 | 716 | < | 398.0 |
| avePPI | 1 | 0.3 | 0.7 | 3.8 | > | 1.1 |
| BAD | 1 | 1.5 | -0.6 | 14.5 | > | 1.2 |
| Kmax | 44.8 | 2.6 | 40.7 | 65.4 | > | 45.7 |
| ISI | 0.1 | 0.8 | -1.4 | 5.5 | > | 0.5 |
