# Supplementary material for: Exploring the Potential of ChatGPT-4 in Predicting Refractive Surgery Categorizations: Comparative Study
Source: JMIR Form Res. 2023 Dec 28;7:e51798. doi: 10.2196/51798 (PMC10784977; doi:10.2196/51798)
Supplement: Multimedia Appendix 13 [file formative_v7i1e51798_app13.docx]

| Categories 1 and 2 | | Categories 2 and 5 | |
| --- | --- | --- | --- |
| pachymetry | -0.174332 | pachymetry | -0.340331 |
| ACD | 0.132466 | ARTmax | -0.300218 |
| sex | -0.117701 | BAD | 0.141738 |
| WtW | 0.108737 | avePPI | 0.122493 |
| age | -0.106124 | sex | -0.098139 |
| ARTmax | -0.104149 | sphere | 0.093726 |
| ISI | -0.094807 | age | -0.059086 |
| avePPI | -0.083695 | Kmax | 0.030648 |
| sphere | -0.08081 | ISI | -0.026772 |
| Kmax | -0.070367 | cylinder | 0.026704 |
| cylinder | -0.069938 | ACD | -0.02326 |
| BAD | -0.045672 | WtW | -0.022996 |
| axis | 0.036772 | axis | 0.010164 |
